# Supplementary material for: The propagation of active-passive interfaces in bacterial swarms
Source: Nat Commun. 2018 Dec 18;9:5373. doi: 10.1038/s41467-018-07781-y (PMC6299137; doi:10.1038/s41467-018-07781-y)
Supplement: Supplementary file 1 — Supplementary Information [file 41467_2018_7781_MOESM1_ESM.pdf]

SUPPLEMENTARY MATERIALS

The propagation of active-passive interfaces in bacterial swarms

Alison E. Patteson[1,2], Arvind Gopinath[3,4] and Paulo E. Arratia[1]

[1]*Department of Mechanical Engineering & Applied Mechanics, University of Pennsylvania, Philadelphia, PA 19104*

[2]*Physics Department, Syracuse University, Syracuse, NY 13244*

[3]*Department of Bioengineering, University of California Merced, CA 95340*

[4]*Health Sciences Research Institute, University of California Merced, CA 95340*

I. SUPPLEMENTARY FIGURES

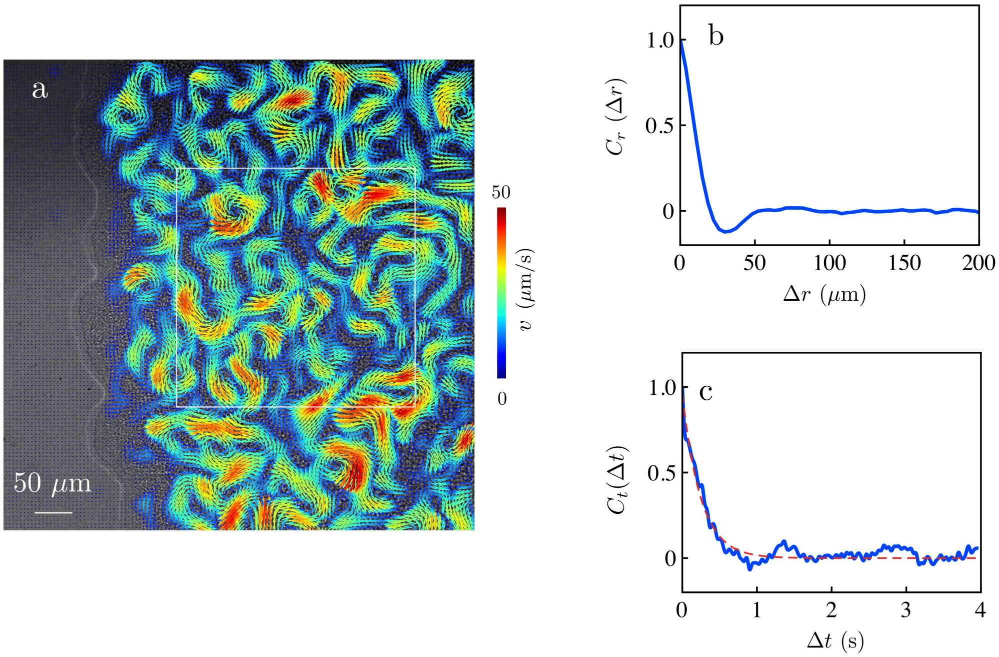

Supplementary Figure 1. Characterization of the bacterial velocity field. (a) A sample snapshot the colony edge overlaid by the bacterial velocity field. The velocity fields are measured using particle image velocimetry (PIV). Experiments are conducted close to the colony edge - in the area inside the white box - where collective motion is observed to be strongest. (b) Spatial and (c) temporal autocorrelations of the bacterial velocity field in its initial state (before exposure to high-intensity light). Measurements are made in the region surrounded by the white box in (a). The data shows that the collective flows in the swarm are correlated over a characteristic length ≈ 20 μm and time ≈ 0.24 s.

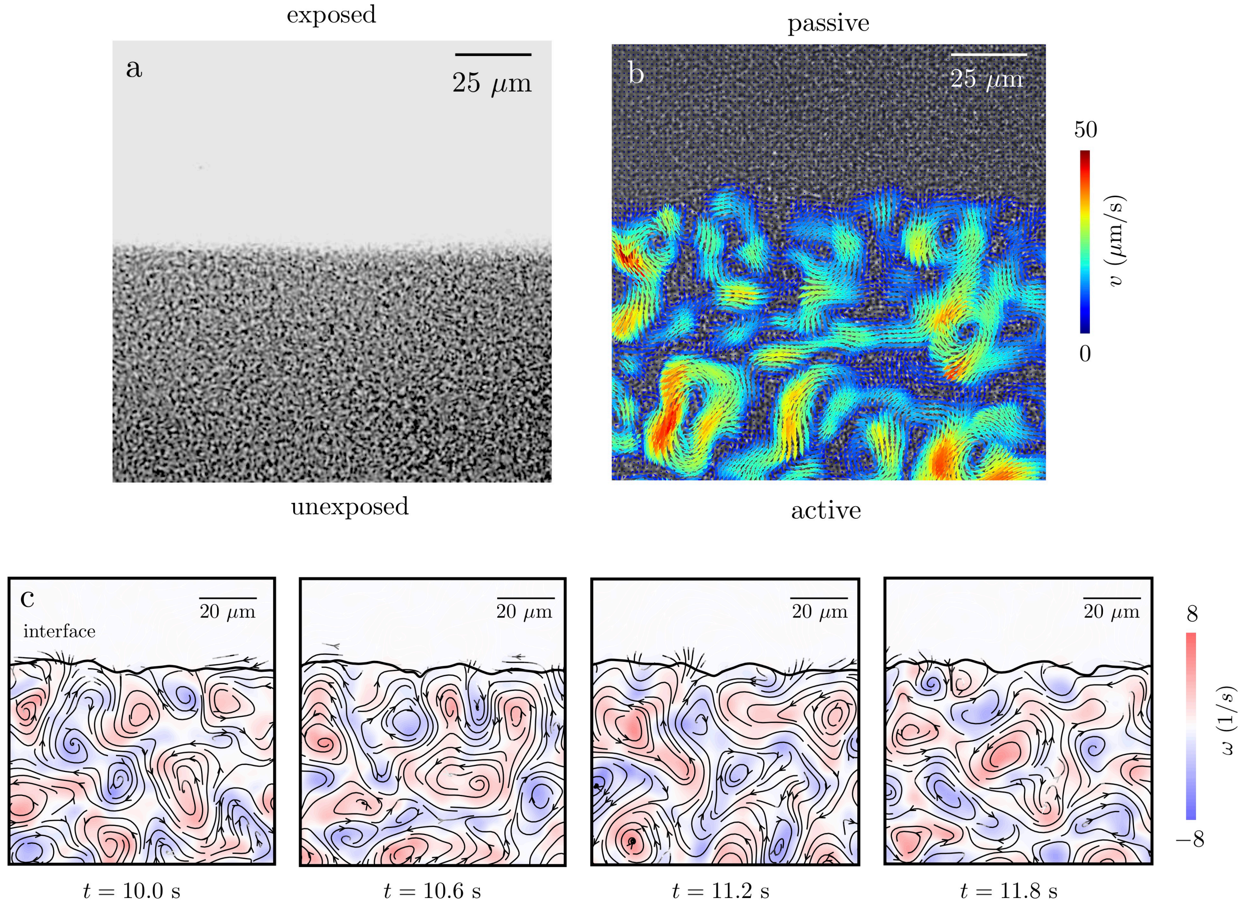

Supplementary Figure 2. Velocity fields from the half-space aperture geometry. (a) An image of the swarm during exposure shows a sub-region of the swarm exposed to a half-plane of high-intensity light. (b) The bacterial velocity field ($t = 10$ s past exposure) shows that at the top (where the swarm was exposed) the bacteria are immobile while at the bottom bacteria continue swarming. (c) A time sequence of the bacterial vorticity field ω and corresponding streamlines near the interface (overlaid black line). Snapshots start $t = 10$ s past exposure. The vortices change over time and clockwise and counterclockwise vortices are often observed aligned next to each other along the interface.

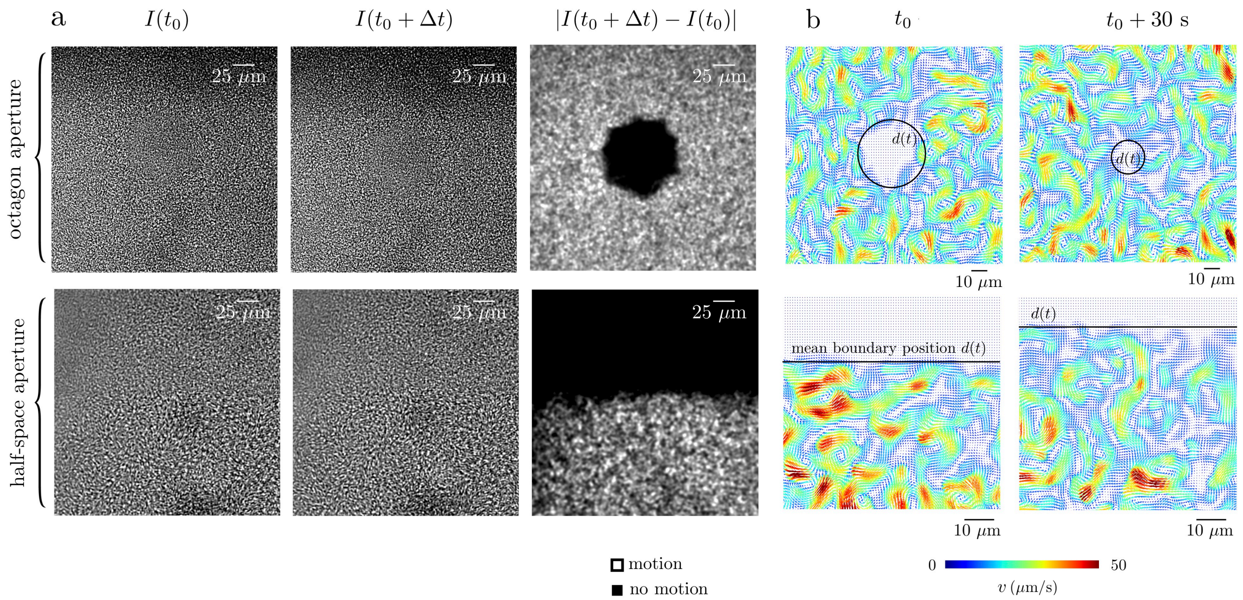

Supplementary Figure 3. Order parameter measurements. (a) Typical images of the swarm taken at $\Delta t = 0.1$ s intervals for the octagonal [O] and half-plane [H] aperture geometries. The individual images do not clearly distinguish the passive phase from the active phase, but fluctuations in the pixel intensity $|\Delta I|$ (rightmost column) do. (b) Sample velocity fields of the octagonal and half-plane aperture geometries shown at 30 second intervals. The black lines correspond to the average interface position d.

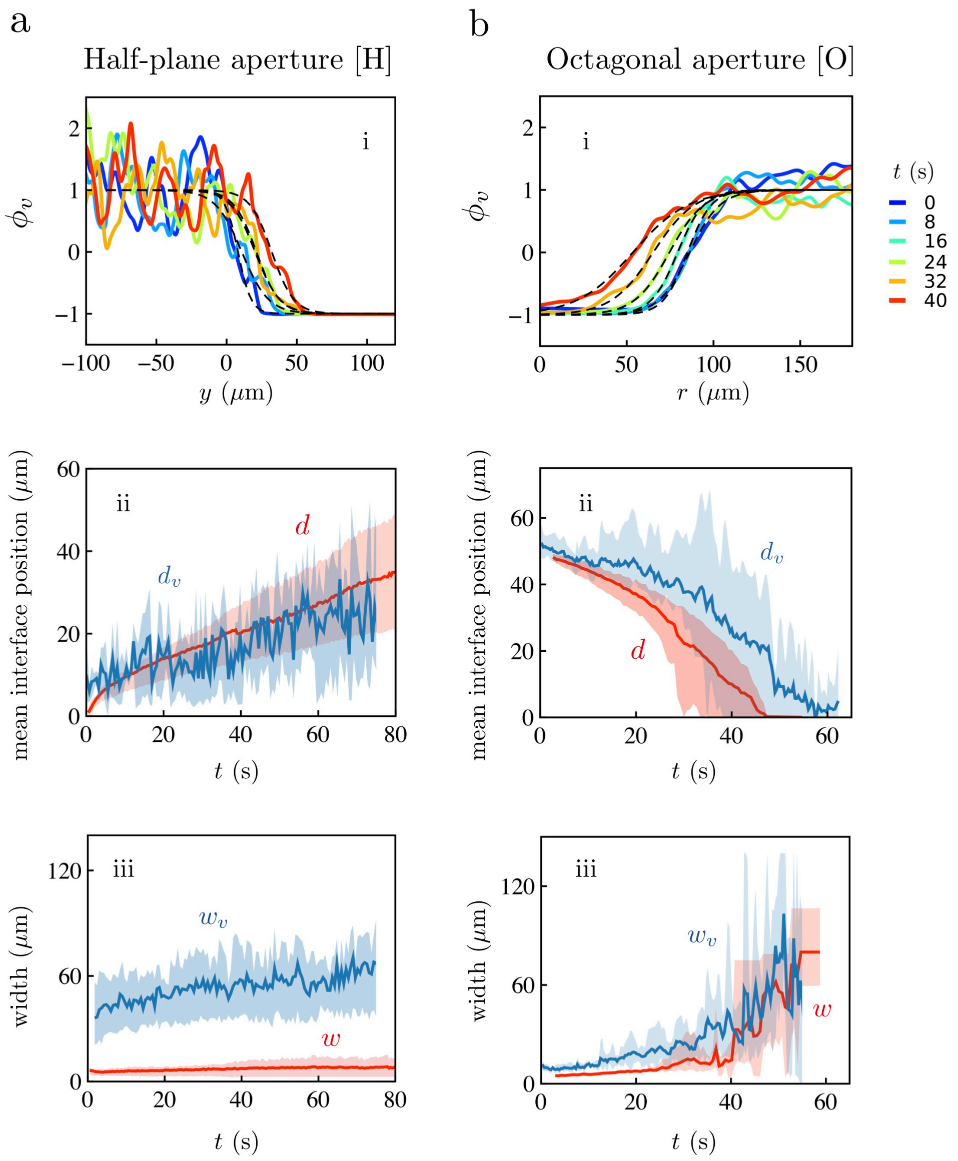

Supplementary Figure 4. The active-passive interface characterized through the bacterial velocity field. Data in (a) corresponds to the half-plane aperture geometry and (b) to the octagonal aperture geometry. (i) One-dimensional phase profile ϕ_v^* (derived from ϕ_v) for the half-plane ([H], a-i) and octagon ([O], b-i) over time. The data are fit to a hyperbolic tangent equation (black dashed line), which corresponds to a spreading propagating interface. (ii & iii) To compare these phase interface profiles with those from the intensity fluctuations (Fig. 2 main text), we plot the mean boundary positions (ii) and interface width (iii) from each over time. The notation d corresponds to ϕ (red) and d_v from ϕ_v (blue). Likewise, $w(t)$ corresponds to ϕ and $w_v(t)$ from ϕ_v. The data is extracted from fits to the phase profiles (i, Eq'n 5 & 6), and results from four independent experiments are combined to obtain the mean, minimum, and maximum values as shown. We find that the interface positions measured from ϕ_v and ϕ are approximately the same whereas estimated widths are larger for ϕ_v (bacterial speed) than for ϕ (intensity fluctuations).

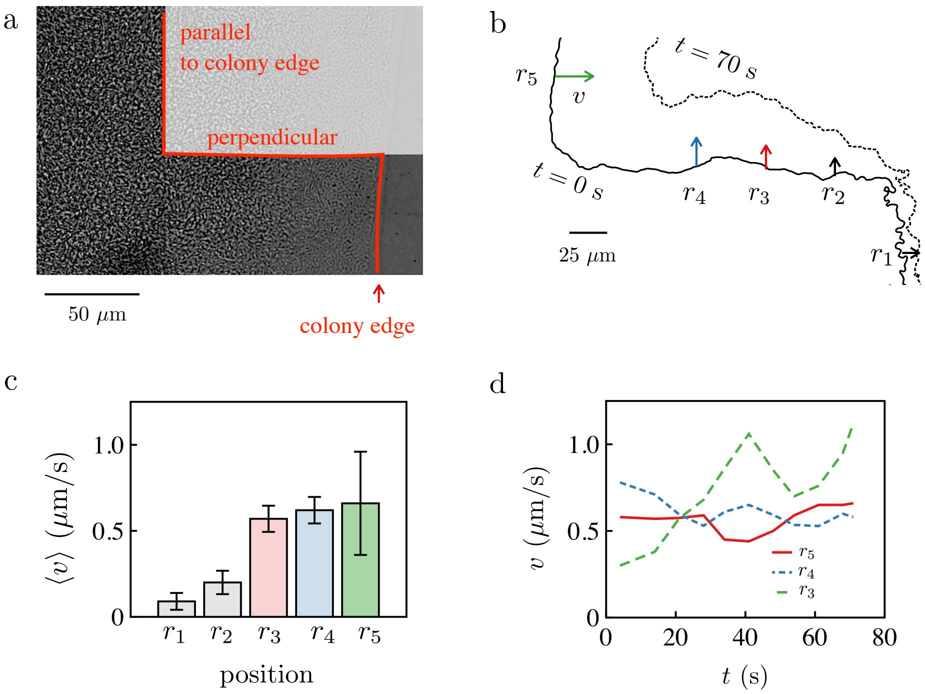

Supplementary Figure 5. Interface erosion dynamics depends on the direction of the expanding colony front. (a) Schematic of a rectangular aperture geometry that creates two active-passive interfaces, one that is parallel to the colony edge and one that is perpendicular. (b) As shown by the active-passive interface (left) and colony edge (right) positions tracked together, the active-passive interface moves more than the expanding colony edge over the 70 second time interval shown. We measure the speed v of the interfaces at the five labeled locations r. (c) The average interface speed is greatest in the bulk of the swarm (r_3, r_4, and r_5) away from the colony edge. Error bars are standard deviation (from gathering over time). (d) Traces of the interface velocity over time show that the interface velocity is approximately constant for locations along the perpendicular interface (r_3 & r_4), while the speed increases for the parallel interface (r_5).

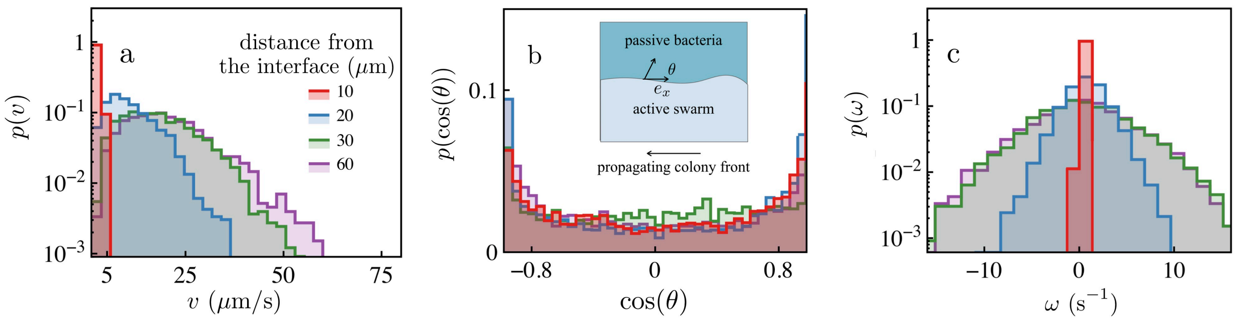

Supplementary Figure 6. Probability distribution functions of (a) the bacterial speed v, (b) the bacterial velocity orientation $\cos(\theta)$ and (c) the flow vorticity ω for varying distances from the active-passive interface (half-space [H] aperture geometry).

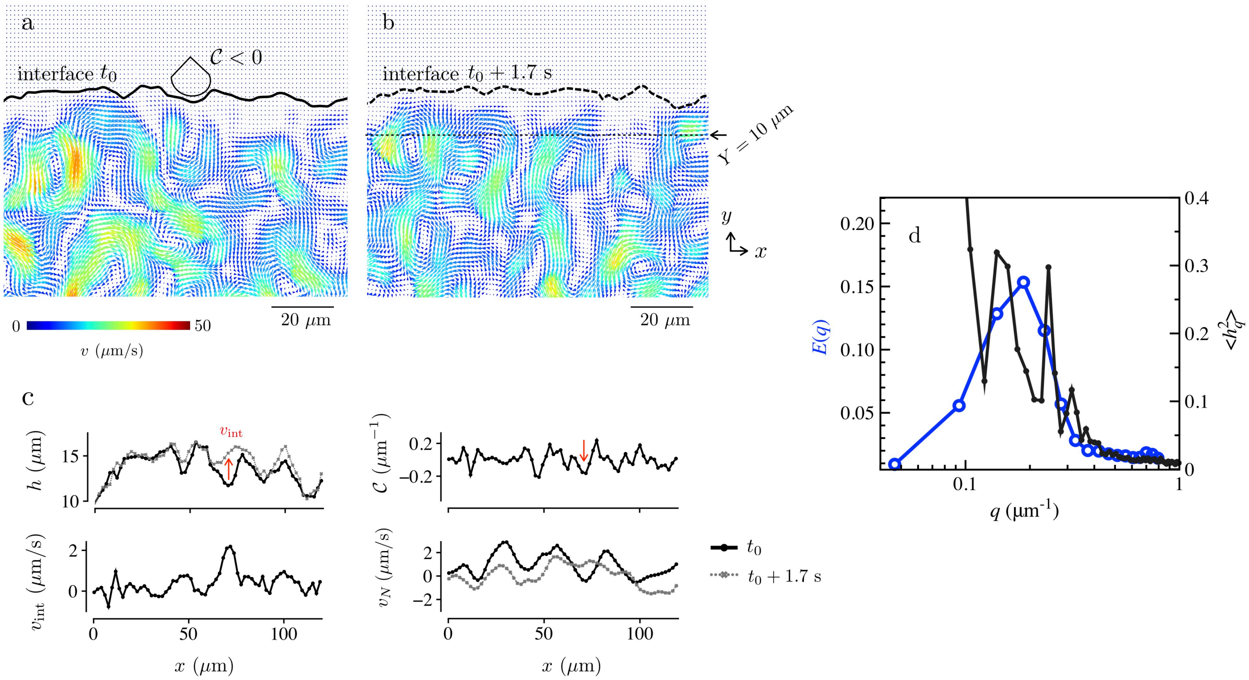

Supplementary Figure 7. Linking the bacterial velocity field to the motion and curvature of the active-passive interface. (a,b) We discern local variations in the interface position and bacterial velocity fields by considering discrete time intervals of $\Delta t = 1.7$ s, which is large enough to distinguish displacements in the interface position $h(x,t)$. (c) For this sample time step, the interface speed ranges from -0.5 to 2 μm/s along the interface; the location of largest interface velocity ($x \approx 75$ μm) corresponds to a location of negative interface curvature $\mathcal{C}$. The bacterial velocity profiles are gathered approximately parallel to the interface at a distance of $Y = 10 \pm 3$ μm away from the mean interface position in the moving coordinate frame $Y = y - d(t)$ (dashed line in (b)); we find that the normal velocity components v_{N} are correlated over the $\Delta t = 1.7$ s time step. (d) Overlay of the energy spectrum of the bacterial flow $E(q)$ and the static structure factor of the interface $|h_q^2|$.

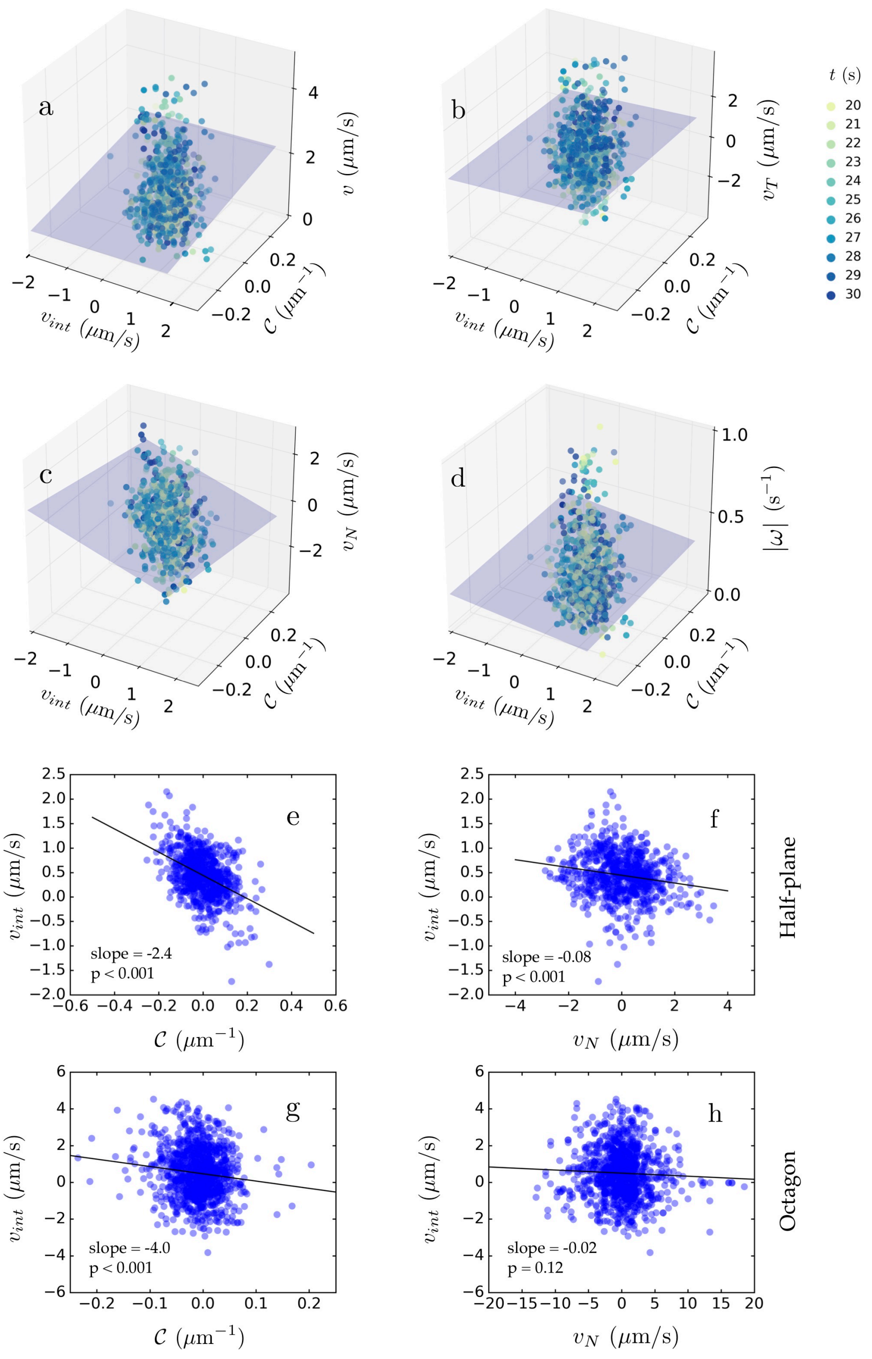

Supplementary Figure 8. (a-d) Scatter plots of local interface speed v_{int} and interface curvature $\mathcal{C}$ versus parameters that characterize the corresponding bacterial flow fields near the interface: the parameters include (a) the bacterial speed v, (b) the tangential velocity component v_{T}, (c) the normal velocity component v_{N}, and (d) the vorticity ω. Our spatiotemporal data collapses unto a plane for v, v_{N}, and ω. The planes are obtained by least-squares fits of the data to a linear form $v_{\mathrm{int}} = a + b\,\mathcal{C} + c\mathcal{X}$, where $\mathcal{X}$ corresponds to v, v_{T}, v_{N}, or ω, respectively. (e-h) Two-dimensional projections of the v_{int}-$\mathcal{C}$-v_{N} plane for the half-space and octagon geometries. The slope from a linear-regression test is presented for each plot and the corresponding p-value from a two-tail t-test with a confidence interval of 95%.

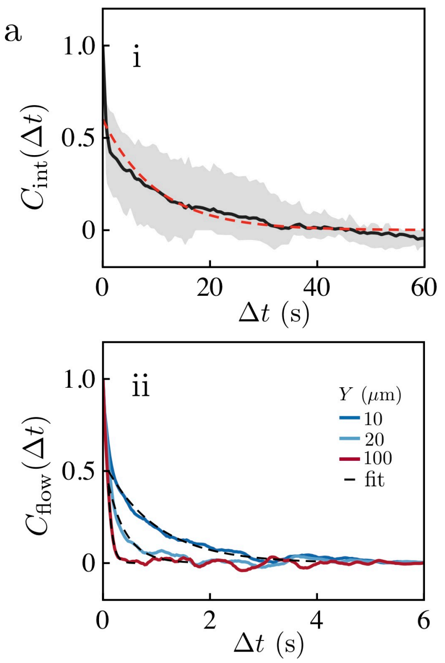

Supplementary Figure 9. Temporal correlations characterizing the interface height and bacterial flow fields. The decay of the interface ($\tau = 16$ s) is significantly slower than that of the flow (<1 sec.).

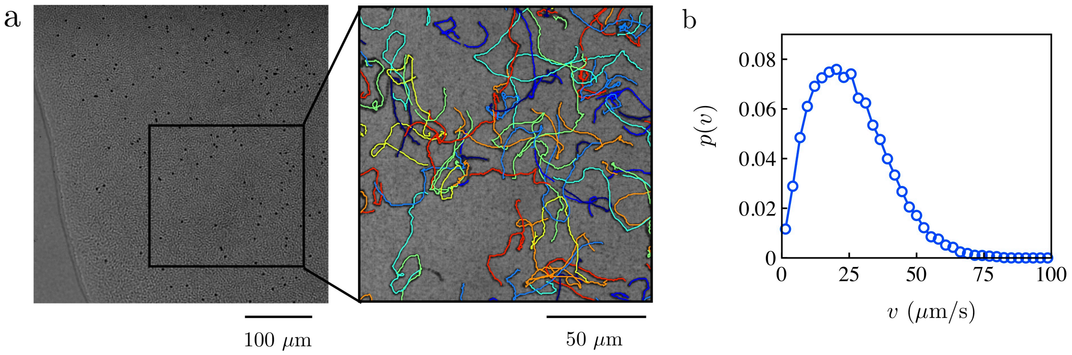

Supplementary Figure 10. (a) An image of the advancing bacterial colony front embedded with 2 μm polystyrene spheres: we use the tracers as probes of the active swarming collective flows in the region of the swarm enclosed by the black box. The particle trajectories are obtained from standard particle tracking techniques. The sample trajectories are shown for a 4 second time interval. (b) We determine the particle speed distribution $p(v)$ by pooling together the particle speed over time for hundreds of particles; the particle speed is defined as the two-dimensional particle displacement over a 1 second time interval, which is long enough to allow tracers to sample multiple vortex structures (characteristic lifetimes of ~ 0.1 second, main text Fig. 3). The particle speed distribution measurement (blue circles) seems to follow a 2D Maxwell-Boltmann distribution (red dashed line), $p(v) = vm(k_B T_{\text{eff}})^{-1}\exp(-mv^2/2k_B T_{\text{eff}})$, where m is the mass of the polystyrene particle, k_B is the Boltzmann constant, and $T_{\text{eff}} \approx 2.2 \times 10^5$ K, approximately 700 times the thermal temperature (293 K). This effective temperature is to be interpreted as a mixture temperature purely due to the energy in the swarming collective flows.

II. SUPPLEMENTARY NOTES

Supplementary Note 1: Details of high intensity light exposure

The effects of high intensity light exposure on the swarming motility of *Serratia marcescens* was explored for a range of exposure times and light intensities. The light source was an unfiltered wide-spectrum mercury lamp. We used standard fluorescence microscope optics to focus the light on selected areas of the bacterial swarm. The shape of the selected area was controlled by the aperture geometry, which was either an octagonal aperture [O] or a half-plane aperture [H]. We varied the light intensity using neutral density filters, and the intensity of light incident on the sample was measured using a spectrophotometer (Thorlabs, PM100D). The response of the bacteria to the light depends on the exposure time and light intensity. We observed three types of bacterial response to the light: (i) always active, (ii) temporarily passive, or (iii) always passive. In case (i), the cells remain motile during light exposure and do not stop moving. In case (ii), the cells stop moving but regain their motility suddenly, typically in the first 5 seconds after the light is switched off. In case (iii), the exposed cells stop moving and do not regain their motility for the duration of the experiment (60 - 300 seconds). We found that for small exposure times (20 - 40 seconds) and weak intensities ($I < 220$ mW at 535 nm) the cells remain always active or are temporarily passivated. For long exposure times ($>$ 60 seconds) and sufficiently high intensities ($I > 220$mW at 535 nm), the bacteria are rendered permanently passive. For our experiments, we choose an exposure time of 60 seconds at intensity $I = 370$ μW (535 nm) to ensure that the exposed bacteria are passive throughout the experiment.

Supplementary Note 2: Bacterial velocity fields from PIV

Bacterial velocity fields were extracted from videos using particle image velocimetry (PIV, PIVLab [1]). Velocity vectors were computed by calculating spatial correlations between successive images of bacteria; the video frame rates were 60 or 125 frames per second, and the velocity fields were sampled at 3 μm spatial intervals. Since we do not visualize the fluid suspending the bacteria, the bacterial velocity fields we extract will generally differ from fluid velocity fields. A sample snapshot of the swarm edge and associated velocity fields before exposure is shown in Supplementary Figure 1(a). The fastest moving cells are observed approximately 100-500 μm from the edge of the advancing colony.

We characterize the initial (pre-exposure) bacterial flow in a 400 μm^2 area, a distance of 100 μm from the edge of the expanding colony (white box, Supplementary Figure 1(a). We calculate the spatial correlation function of the bacterial velocity $C_v(\Delta r)$ and the temporal correlation $C_t(\Delta t)$ from the PIV data using the

relationships

$$C_r(\Delta r) = \Big\langle \frac{\mathbf{v}(r_0)\cdot\mathbf{v}(r_0+\Delta r)}{|\mathbf{v}(r_0)|^2} \Big\rangle, \tag{1}$$

$$C_t(\Delta t) = \Big\langle \frac{\mathbf{v}(t_0)\cdot\mathbf{v}(t_0+\Delta t)}{|\mathbf{v}(t_0)|^2} \Big\rangle. \tag{2}$$

In supplementary equations 1 and 2, the angular brackets denote averages over reference positions r_0 (within 400 μm^2 white box, Supplementary Figure 1(a) and times t_0 (in a 30 sec. time interval).

Figure 1(b) shows the radial correlation function $C_r(\Delta r)$ of the bacterial flow field. The data reflects the large-scale collective motions in the bacterial flow, which lead to correlations inside a bacterial vortex and anti-correlations outside, corresponding to a peak and minimum in C_r. For Δr less than a vortex length, the bacterial velocities are correlated positively, $C_r > 0$. As Δr increases, the function C_r decays, crosses zero, and is negative for 21 μm $< \Delta r <$ 32 μm. This separation range correlates with neighboring vortices being anti-correlated with opposite vorticity (counterclockwise CCW, and clockwise CW). For $\Delta r > 32$ μm, velocity fields become progressively uncorrelated and $C_r \to 0$ again. Extracting the value of Δr at which the correlation function first crosses zero, we estimate that the vortices are approximately $\lambda_c \approx 20$ μm.

The dynamics of the vortical flow structures are characterized by examining the temporal correlation function C_t shown in Supplementary Figure 1(c). The initial decay is captured reasonably well by the form $C_t(\Delta t) = \exp(-\Delta t/\tau)$ with $\tau \approx 0.24$ s interpreted as the typical lifetime of a vortex. The oscillations in C_t indicate that the vortical flows fluctuate between clockwise and counter-clockwise directions approximately every 0.8 seconds.

Flow at the interface

We next examine the velocity fields near the interfacial region that separates the swarming active phase region from the immobile passive phase. Supplementary Figure 2(a) is an image of the swarm during exposure with the [H] aperture, and Supplementary Figure 2(b) displays the corresponding bacterial velocity field after exposure.

The top half of the image is the exposed domain; as expected the velocity magnitudes are zero in this region. In contrast, the bottom half is unexposed part, and bacteria continue to exhibit strong motility and collective motions, maintaining speeds up to 50 μm/s. From visual inspection, we find that the interface

appears rough (with small scale fluctuations) at length scales of the order of bacterial lengths but smooth and flat at larger length scales. We point out that unlike the interface generated by exposure through the [O] aperture (Figs 1 & 2, main text), the interface generated the [H] aperture has no imposed corners.

To phenomenologically characterize the spatiotemporal features of the collective flow near the interphase boundary, we zoom in on a part of the domain and plot the vorticity field at 0.6 s intervals as shown in Supplementary Figure 2(c). The swarming flows are quasi two dimensional (the layer being thin), and the vortical structures are well captured by the two-dimensional vorticity $\omega = \partial_x v_y - \partial_y v_x$. The Cartesian components of the velocity vectors v_x and v_y are extracted from PIV data. Supplementary Figure 2(c) suggests that vortices align along the interface, arrayed in an antisymmetric (clockwise/counterclockwise) pattern. Over time, the individual vortices appear to move, split, and merge together. These dynamic vortices etch the interface into cusps and valleys and continuously impact its dissolution.

Supplementary Note 3: Defining and tracking the interface

Order parameters track the interface

The active bi-phasic system we study differs from classical bi-phasic passive systems, such as a solidifying melt or melting ice, in many ways. Perhaps the most noteworthy of these is the way in which the interfacial region is continuously eroded and remodeled by the extraction and convective redistribution of paralyzed, exposed bacteria from the passive phase by emergent self-organized flows generated in the active phase. Note that while passive bacteria enter the active phase as they are convected away from the interface, their fraction far from the interface is expected to be small since the overall (initial) size of the passive phase is small compared to the total swarming area.

Nevertheless, direct observation of the interface between the active and passive phases and results from PIV illustrating a *mixing region* between the two phases suggests that the inter-phase boundary may be represented as a *diffuse interface with a finite thickness* with the density of active motile bacteria varying very sharply across a *boundary layer thickness*. A mathematically defined diffuse boundary may then be obtained from the phase field profiles using order parameters as done in previous investigations involving interface phenomena [2]. To test if such a description may prove useful in our active system, we utilize two independent scalar order parameters, one based on intensity fluctuations and the other based on the bacterial velocity, to track and characterize the interface.

1. Order parameter from intensity fluctuations

We first use a dynamic order parameter ϕ based on pixel intensity fluctuations measured from successive images of the swarm. This approach assumes that fluctuations in image intensity correlate with fluctuations in bacterial density. This assumption forms the basis of differential dynamic microscopy (DDM) [3–9], which is an image analysis technique also used to characterize the motility of particles or cells from video images.

Here, we define intensity fluctuations as $|\Delta I(\mathbf{r}, t, \Delta t)| = |I(\mathbf{r}, t + \Delta t) - I(\mathbf{r}, t)|$, where $I(\mathbf{r}, t)$ is the image intensity at pixel position $\mathbf{r}$ (in 2 dimensions) at time t and Δt is the time step [4, 6]. We choose $\Delta t = 0.1$ s, which corresponds to the time in which a swarming *Serratia* cell swimming at 20-50 μm/s moves roughly a body distance. We checked that our results do not depend on the exact choice of Δt in the range 0.05 s $< \Delta t <$ 0.3 s. Supplementary Figure 3 shows sample images of the swarm at 0.1 time intervals and the corresponding map in intensity fluctuations $|\Delta I(\mathbf{r}, t, \Delta t)|$ for both the half-space and octagonal apertures. Dark regions of the $|\Delta I(\mathbf{r}, t, \Delta t)|$ maps represent low pixel fluctuations and correspond to domains of immotile cells; bright regions represent larger fluctuations and correspond to moving cells. We find that the mean intensity fluctuations far from the boundary remain steady over time for the active ($|\Delta I_A(t)|$) and passive phases ($|\Delta I_P(t)|$) (data not shown), and we define the order parameter ϕ at each time t as

$$\phi(\mathbf{r}, t) = \frac{2|\Delta I(\mathbf{r}, t)| - |\Delta I_A(t)| - |\Delta I_P(t)|}{|\Delta I_A(t)| - |\Delta I_P(t)|}. \tag{3}$$

To reduce noise in the system (due to pixel resolution, short-range fluctuations, and background light fluctuations), we filter the pixel-wise calculated order parameter by smoothing the data over 3 x 3 μm^2 areas.

The averaged ϕ-field satisfies $-1 \leq \phi \leq +1$, with -1 corresponding to a completely passive phase and 1 corresponding to a completely active phase (Fig. 2, main text). The locus of points set by $\phi = 0$ is defined as the interface separating the active and passive phases.

2. Order parameter from PIV velocity fields

Alternately, active and passive domains of the swarm can also be identified by tracking the bacterial velocity fields $\mathbf{v}(\mathbf{r}, t)$. As evident from Supplementary Figure 2(b), the passive phase is readily identified as the region where the macroscopic PIV-derived bacterial speeds are zero. We note that there are locations in the active phase that have instantaneous velocities near zero; however spatial averages ($>$ 10 μm) of the velocities or temporal averages (over $\sim$ 1 s) discern between the fully active and fully passive phases. We

use the velocity fields to define a second order parameter ϕ_v map as

$$\phi_v(\mathbf{r},t) = \frac{2v^2(\mathbf{r},t) - v_A^2 - v_P^2}{v_A^2 - v_P^2} \tag{4}$$

where v_A and v_P are the average velocity of the active and passive phases far from the interface and $\mathbf{r}$ is the position of the velocity vectors (sampled at 3 μm intervals). Again, $-1 \leq \phi_v \leq +1$, with -1 corresponding to a completely passive phase and 1 corresponding to the active phase.

3. Phase fields ϕ and ϕ_v provide interface position and width

To compare the bacterial velocity phase fields with those from intensity fluctuations, we determine spatially-average phase profiles from each. The two-dimensional phase fields (ϕ and ϕ_v) may be expressed in Cartesian (x, y) or polar (r, θ) coordinates. For experiments using the [H] aperture, we average $\phi(x, y, t)$ over x, the appropriate arc-length coordinate for this geometry to obtain a one-dimensional phase profile $\phi^*(y, t)$. For experiments using the [O] aperture, we exploit the initially discrete symmetry and azimuthally average the order parameter to obtain the one-dimensional radially dependent field $\phi^*(r, t)$. The same procedure extended to the velocity based fields yields the one dimensional descriptions $\phi_v^*(y, t)$ and $\phi_v^*(r, t)$. We find that profiles of ϕ_v^* (Supplementary Figure 4) are qualitatively similar to ϕ^* (Fig. 2, main text). In particular, both phase-fields follow a hyperbolic tangent form over a significant part of the time the interfaces are tracked. We therefore obtain the (mean) interface position $(d(t), d_v(t))$ and (average) interface width $(w(t), w_v(t))$ by fitting the phase profiles at each time t to either

$$\phi^* = \tanh\left[\frac{y - d(t)}{w(t)}\right], \quad \phi_v^* = \tanh\left[\frac{y - d_v(t)}{w_v(t)}\right], \tag{5}$$

for experiments exposed using the [H] aperture geometries or

$$\phi^* = \tanh\left[\frac{r - d(t)}{w(t)}\right], \quad \phi_v^* = \tanh\left[\frac{r - d_v(t)}{w_v(t)}\right], \tag{6}$$

for experiments with the [O] aperture.

Comparing the interface positions and widths obtained by the two order parameters (ϕ and ϕ_v), we find that the mean interface position does not depend on the choice of the order parameter - evidenced by the observation that $d(t)$ and $d_v(t)$ match (Supplementary Figure 4). The interface thickness however does depend on the order parameter and is larger when computed from ϕ_v than ϕ. For instance, in the [H] aperture case, $w \approx 7$ μm while $w_v \approx 40$ μm. This disparity may arise because velocity fields vary

over lengths comparable to a vortex size (≈ 20 μm, Fig. 3 main text), whereas intensity fluctuations vary over the length scale of a bacterium ($\approx$ 5-10 μm). Despite the difference in magnitudes, w and w_v exhibit qualitatively similar temporal variations for both the octagonal and half-space aperture geometries. For the rest of our analysis, we chose to use ϕ (and by extension ϕ^*) rather than ϕ_v (see main text) as intensity fluctuations measurements are better resolved spatially than the velocity fields.

4. Choosing initial alignment of exposed region relative to colony edge

Given that the bacterial colony expands radially out from the inoculation cite, we also investigated how the location and alignment of the active-passive interface relative to the bacterial colony edge impacts the dissolution trends. We focus on two questions: (1) how does the active-passive interface velocity depend on the distance from the colony edge and (2) how does the direction in which the colony expands (relative to the interface) influence the active/passive interface velocity?

To answer these questions, we used an aperture that resulted in an exposure pattern with two mutually perpendicular straight edges as shown in Supplementary Figure 5(a). The exposure is aligned so that one edge lies perpendicular to the colony edge and one edge lies parallel. We track each edge of the active-passive interface along with the expanding edge of the colony. As shown by the boundary positions in Supplementary Figure 5(a), the parallel and perpendicular active-passive interfaces moved significantly more than the colony edge for the $\Delta t = 70$ s time interval shown.

Next, we measure the interface speed (normal components of the surface motion) at the five locations labeled in Supplementary Figure 5(b). The speed is defined by the displacement over a time interval of 5 seconds. Here, the edge of the colony (r_1, Supplementary Figure 5(c)) expands at an average speed of $\approx$ 0.1 μm/s. The average speed of the active-passive interface is larger than the colony front and increases from 0.2 μm/s to 0.6 μm/s as one moves away from the bacterial colony front into the swarm (r_3,r_4,r_5, Supplementary Figure 5(b) and 5(c)). This behavior may be due to changes in the swarming velocity of the cells observed in Fig. 1 (main text): cells at the edge of the expanding colony move slower compared to bacteria within the swarm (50 to 500 μm from the colony edge). Previous investigations involving swarming bacteria [12] showed that cells at the edge of the colony frequently stall and even stop moving, resulting in an overabundance of slow-moving cells at the edge. Our measured dependence of interface speed with colony edge distance suggests that the active-passive interface speed may be limited by the mean bacterial swarming velocity.

We next consider the orientation of the active-passive interface relative to the colony edge. We find that the temporal dynamics of the interface speed depends on this orientation (Supplementary Figure 5(d)). The

perpendicular interface speed is reasonably constant over time (as shown for r_3 and r_4); however the parallel interface (r_5) has a time-dependent erosion rate, the speed increasing from 0.3 μm/s to 1.2 μm/s over an interval of 70 seconds past exposure. We conjecture that the parallel interface may block the expansion of the swarm, and the observed increase in velocity over time could be due to an accumulation of actively swarming bacteria at the interface; if an increase in bacterial density increases the interface speed, then an accumulation of bacteria at this interface could lead to an increasing interface speed over time. Based on the results, we conducted our experiments in the range of 100 to 500 μm from the colony edge to avoid spatial variations in bacterial swarming velocity and we align the flat active-passive interfaces perpendicularly to the swarm edge so that the interface velocity is constant over time.

Supplementary Note 4: Statistics of flow near the interface

A. Velocity and vorticity fields

We now describe the phenomenological features of the bacterial flow as one moves away from the interphase boundary. We focus on experiments conducted using the [H] aperture geometry and define a coordinate variable $Y = d(t) - y$ that measures the normal distance perpendicular to the instantaneous mean position of the interface, $d(t)$. Note that larger positive values of Y correspond deeper into the active phase.

PIV data is used to calculate three time-averaged probability distribution functions for varying distances Y: these functions include (i) $p(v)$, the distribution of bacterial speed, (ii) $p(\cos(\theta))$, the distribution of velocity orientation, and (iii) $p(\omega)$, the distribution of bacterial vorticity field ω. We denote the velocity orientation θ with respect to the active-passive interface as shown in the schematic (inset). The vorticity ω is defined as $\omega = \partial_x v_y - \partial_y v_x$ with v_x and v_y being the PIV velocity components.

These distributions are shown in Supplementary Figure 6. For the bacterial speed v, the probable magnitudes increase with Y for $Y < 40$ μm. For $Y > 40$ μm, however, $p(v)$ does not vary significantly with Y. The distribution $p(\cos(\theta, Y))$ exhibits clear peaks in orientation at $\theta = \pm\pi$. Since this orientation corresponds to the direction of the moving colony front and this bias occurs at all distances from the interface Y, we infer that it is due to the expansion of the swarm and not the presence of the active-passive interface. In the case of vorticity, the probability distributions extend to higher magnitudes with increasing Y, indicating that vorticity is stronger away from the interface. Because the $p(\omega)$ distributions are symmetric at all Y, there is no bias in clockwise or counterclockwise vortices at the interface or in the bulk. Summarizing the results in Supplementary Figure 5, we deduce that the presence of the interface results in a decrease

in the magnitudes of the bacterial velocity and vorticity but does not change the overall shape of the flow distributions relative to the bulk active flow.

Supplementary Note 5. Curvature and flow

A. Sampling interface and flow data

Since the active-passive interface is continuously deformed and eroded by the swarming active phase, we expect that the erosion speed $v_{\rm int}$ correlates the local bacterial velocity. Appealing to the classical interface propagation phenomena in active two-phase systems, we hypothesize that interface curvature may play a role in this active system as well, influencing the local erosion rate and bacterial flow. Spatiotemporal correlations connecting interface structure and flow statistics are therefore expected to appear at finite length and time scales.

To test our hypothesis, we sample the interface and flow in discrete steps, described as follows for the half-space aperture [H] experiments, which are not limited by finite-time dissolution effects. Interface positions, curvatures and interface velocity were determined as follows. Given the map of points $(x_{\rm I}, y_{\rm I})$ that define the interface and an arc-length parameter s measuring distance along the interface, the (raw) interface curvature $\mathcal{C}$ was calculated using the implicit relationship

$$\mathcal{C} = \frac{x'_{\rm I} y''_{\rm I} - y'_{\rm I} x''_{\rm I}}{(x'^2_{\rm I} + y'^2_{\rm I})^{3/2}} \tag{7}$$

where the prime denotes the derivative along the arc length. The curvature $\mathcal{C}$ to be negative for the passive region with a concave boundary (Supplementary Figure 7(a)) and average $\mathcal{C}$ over lateral lengths 3 μm to remove small scale fluctuations. Note that this length scale retains features that are of the order of bacterial lengths while eliminating fluctuations over scales of the order of the bacterial thickness. For the interface given by the Monge approximation applied to the results for experiments with the [H] aperture where the mean height is a straight line, equation (8) simplifies further to yield the curvature as a function of the height $h(x, t)$ using standard geometry relating the curvature to variations in the arc-length in two dimensions. Note that this is possible since to leading order the mean interface is flat, and overhangs are neglected since we observe them very rarely. To smooth over individual bacteria, we average the interface position over a typical bacterial length, 5 μm. The local interface velocity $v_{\rm int}$ at each time t quantifies the erosion rate of the interface and is normal to the tangent vector. Here, we define the local interface velocity as

$$v_{\rm int}(x, t, \Delta t) = \frac{h(x, t + \Delta t) - h(x, t)}{\Delta t}. \tag{8}$$

We choose $\Delta t = 1.7$ s, which is large enough to resolve variations in the interfacial position.

Supplementary Figure 7 displays typical bacterial velocity field data overlaying the interface boundary position. Here, the bacterial velocity is from PIV data, and the boundary position is determined by the phase-field order parameter field ($\phi = 0$). For the sample data shown in Supplementary Figure 7(c), we observe that the interface velocity appears to strongly correlate with $\mathcal{C}$. For instance, the maximum velocity occurs in a region of the interface with an initially large negative curvature (at $x \approx 65$ μm); yet, the interface velocity is negligible where the initial position has large positive curvature (at $x = 75$ μm). These observations suggest that regions of the interface with negative curvature may erode differently than regions with positive curvature.

To understand the long time erosion characteristics, we compare the interface speed and curvature to features of the bacterial flow, specifically the velocity magnitude $|v|$, the tangential velocity component $v_{\mathrm{T}}(= v_x)$, the normal velocity component $v_{\mathrm{N}}(= v_y)$, and vorticity ω. Because bacterial velocities are small at the interface ($Y = 0$ μm) and difficult to resolve, we use bacteria velocities in the range $7 < Y < 13$ μm as a measure of the flow along the interface, noting that this region is less than a vortex size from the interface (Fig. 2, main text). We also note that to leading order the mean interface geometry (at macroscopic length scales) is flat with curvature and roughness fluctuations superimposed on this underlying flat surface. Supplementary Figure 7(c) (bottom right tile) shows two sample profiles of the bacterial flow for the normal velocity component v_{N} (for example) separated by $\Delta t = 1.7$ s (the time interval used to define the interface velocity). The two velocity profiles are correlated as expected for a correlation time of 0.8 s (main text, Fig. 3). This observation allows us to use only the bacterial velocity at the start of the interval when comparing the flow to the interface velocity v_{int}. (We also choose the interface curvature profile corresponding to the start of the time interval, noting that the interface structure changes slowly compared to the flow (main text, Fig. 3).) Finally, the interface curvature and interface speed are interpolated at 3 μm intervals to match the bacteria velocity profile data extracted from the PIV fields and correlations among the variables over time are tested for as described in the following section.

B. Effective boundary conditions

We analyze the v_{int}-$\mathcal{C}$-v data and test if an *active* version of the classical extended Gibbs-Thomson-Stefan relationship can describe the erosion of the interface. We explore correlations among the interface speed v_{int}, interface curvature $\mathcal{C}$, and bacterial velocity field $\mathbf{v}$ (in the vicinity of the interface) based on our hypothesis (main text, Methods and Materials) that, to leading order, local interface velocity is linearly correlated with the local curvature of the interface and local velocities (or equivalently vorticity). Thus we

write

$$v_{\text{int}} = a_1 + a_2\mathcal{C} + a_3\mathcal{X} \quad (9)$$

where the variable $\mathcal{X}$ - as yet undetermined - factors in bacterial activity and the effects of the vortical flows in shaping and deforming the surface. Note that a_1 defines a mean erosion of the interface in the absence of curvature and the collective motion of the swarm and is likely set by the self-propulsive speeds of individual bacteria and orientational effects of the passive phase. It should be emphasized here that by local we mean over length scales that are large compared to single bacteria but small compared to the scale of the vortices in the swarming phase. Consistent with our hypothesis that the vortical nature of the flow drives the dissolution, we used the bacteria speed $|v|$, v_{N} (normal component of the bacteria velocity), v_{T} (tangential component), and bacteria flow vorticity ω as possible stand-ins for $\mathcal{X}$. The fidelity of the fit to the data was tested using statistical measures for each of these chosen variables.

Supplementary Figure 8 contains sample scatter plots of v_{eff}-$\mathcal{C}$-$\mathcal{X}$ data, which is shown for an experiment using an [H] aperture geometry. The data is gathered at 3 μm intervals along the interface and at 1 second time intervals for 10 s $< t <$ 40 s past the light exposure to ensure that the interface dynamics are quasi-steady and to avoid transient states at the beginning of the experiment. We find that the 3D scatter plots collapse reasonably well onto a plane, consistent with equation (9), for v (Supplementary Figure 8(a)), v_{N} (Supplementary Figure 8(c), and ω (Supplementary Figure **??**(d)). The collapse is not as striking for the tangential velocity component v_{T} of the bacterial flow (Supplementary Figure 8(b)) for this geometry. The collapse is strongest for v_{N}, which is the only case to bear a statistically significant correlation ($p < 0.05$) among all three variables. This best fit to the planar form is given for v_{N} by the constants $a_1 = 0.4$ μm/s, $a_2 = -2.3$ μm^2/s and $a_3 = -2.0$, thereby quantifying the synergetic interactions between the interface and the collective flow.

REFERENCES

[1] Thielicke W., Stamhuis E., PIVlab-towards user-friendly, affordable and accurate digital particle image velocimetry in MATLAB. *Journal of Open Research Software* 2, 1 (2014).

[2] Gopinath A., Armstrong RC., Brown RA., J Cryst Growth 291 (1), 272 (2006).

[3] Croccolo F. *et. al.*, Effect of Gravity on the Dynamics of Non-equilibrium Fluctuations in a Free Diffusion Experiment. *Annals of the New York Academy of Sciences* 1077, 365 (2006).

[4] Cerbino R, Trappe V., Scattering information obtained by optical microscopy: Differential dynamic microscopy and beyond. *Phy. Rev. E* 80, 031403 (2008).

[5] Giavazzi F. *et. al.*, Differential Dynamic Microscopy: Probing Wave Vector Dependent Dynamics with a Microscope. *Phys. Rev. Lett.* 100, 188102 (2009).

[6] Wilson LG. *et. al.*, Differential dynamic microscopy of bacterial motility. *Phy. Rev. Lett.* 106, 018101 (2011).

[7] Martinez VA *et. al.*, Differential Dynamic Microscopy: A High-Throughput Method for Characterizing the Motility of Microorganisms. *Biophys. J.* 103, 1637 (2012).

[8] Lu PJ *et. al.*, Characterizing Concentrated, Multiply Scattering, and Actively Driven Fluorescent Systems with Confocal Differential Dynamic Microscopy. *Phys. Rev. Lett.* 108, 218103 (2012).

[9] Martinez VA *et. al.*, Fast, high-throughput measurement of collective behaviour in a bacterial population. *Interface* 11, 20140486 (2014).

[10] Frisch U. Turbulence., *Cambridge University Press* Cambridge, UK (2004).

[11] Wensink HH, *et al*., Meso-scale turbulence in living fluids. *PNAS* 109, 14308 (2012).

[12] Darnton NC, *et al.*. Dynamics of bacterial swarming. *Biophys. J.* 98, 2082-2090 (2010).
