# Supplementary material for: The propagation of active-passive interfaces in bacterial swarms
Source: Nat Commun. 2018 Dec 18;9:5373. doi: 10.1038/s41467-018-07781-y (PMC6299137; doi:10.1038/s41467-018-07781-y)
Supplement: Supplementary file 2 — Description of Additional Supplementary Files [file 41467_2018_7781_MOESM2_ESM.pdf]

Description of Additional Supplementary Files

Supplementary Movie 1
Description: Collective motion of the swarm
A zoomed-in video at the colony edge reveals the dynamic collective motion of bacteria. Scale bar, 20 um. Video played at 0.4x time. Taken with 20X, 0.45 NA objective.

Supplementary Movie 2
Description: Growth of passive domain and dissolution
A sample video of the quenching the swarm with high-intensity light. The light exposure first slows the bacteria down before a region of passive cells forms near the center of the exposure. Eventually, the passive region reaches a quasi-equilibrium state with a finite size less slightly less than the exposure region. When the light is switched off, the surrounding active swarm erodes the passive phase. Cells within the passive phase remain immobile. Here, the exposure time is 150 seconds, and the light intensity is I = 180 uW at 535 nm. Scale bar, 40 um. Video played at 8.2x time. Taken with a 10X, 0.3 NA objective.

Supplementary Movie 3
Description: Collective motion of the swarm around the passive domain
A zoomed-out view of a passive domain in the swarming edge of the bacterial colony. The passive phase is created in the active swarming region of the colony: cells at the outermost edge of the propagating colony front and very far from the edge (>500 um) do not exhibit strong collective motility. The velocity vectors gathered from PIV are overlaid on the video: blue, 0 um/s; red, 50 um/s. Scale bar, 40 um. Video played at 0.4X time. Taken with 20X, 0.45 NA objective.

Supplementary Movie 4
Description: Time lapse of dissolution process.
The active swarming bacteria interact with the passive phase through the boundary. Over time, passive particles are convected into the active phase and the boundary propagates radially inward. Small pockets of active cells can be seen penetrating passive phase (at 00:03), broadening the active-passive interface thickness. Video features 5 second clips; taken at approximately 15 seconds apart. Scale bar, 20 microns. Video played at 0.4x time. Taken with 20X, 0.45 NA objective.

Supplementary Movie 5
Description: Collective flows at the interface with the half-space [H] aperture
A sample video of the flat active-passive interface. The velocity vectors gathered from PIV are overlaid on the video: blue, 0 um/s; red, 50 um/s. Scale bar, 10 um. Video played at 6.5X time. Taken with 63X, 0.7 NA objective.

Supplementary Movie 6
Description: Tracer particle motion at the interface
Polystyrene spheres (2 um) serve as tracer particles of the flow. Trapped particles in the interface first wiggle and fluctuate when the active-passive interface before they are swept away in active collective flows. Scale bar, 15 um. Video played at 0.5x time. Taken with 20X, 0.45 NA objective.
